# Diagnostic, Prognostic, and Therapeutic Roles of Water-Soluble Contrast in Adhesive Small Bowel Obstruction: A PRISMA-Guided Narrative Systematic Review

**DOI:** 10.7759/cureus.113443

**Published:** 2026-07-27

**Authors:** Puneet S Gill, Daniela Yepez

**Affiliations:** 1 Surgery, State University of New York (SUNY) Downstate Health Sciences University, Brooklyn, USA; 2 Internal Medicine, New York University (NYU) Grossman Long Island School of Medicine, Patchogue, USA

**Keywords:** adhesive small bowel obstruction, adhesive small bowel obstruction (asbo), contrast challenge, diatrizoate, gastrografin, nonoperative management, small bowel obstruction (sbo), urografin, water-soluble contrast

## Abstract

Adhesive small bowel obstruction (ASBO) is a common surgical emergency, most frequently caused by postoperative adhesions. Although many patients improve with bowel rest, nasogastric decompression, intravenous fluids, and electrolyte correction, delayed operative intervention in patients unlikely to resolve nonoperatively can increase morbidity. Water-soluble contrast (WSC) agents such as Gastrografin, Urografin, and related diatrizoate preparations have been proposed as diagnostic, prognostic, and potentially therapeutic adjuncts. In this article, we review the available clinical evidence on the use of WSC in adult adhesive or postoperative small bowel obstruction (SBO) and examine whether its primary role is diagnostic and prognostic rather than therapeutic. A structured narrative review was conducted in accordance with PRISMA guidelines, using studies identified through PubMed, Excerpta Medica database (Embase), and manual searches. Eligible studies included adult patients with adhesive, postoperative, or non-strangulated SBO who received oral or nasogastric WSC and reported clinical outcomes such as passage of contrast, obstruction resolution, operative intervention, hospital length of stay, or complications. Randomized trials, prospective observational studies, retrospective cohort studies, and protocol evaluations were included. Due to differences in study design, patient populations, contrast protocols, and outcome definitions, the findings were synthesized narratively. Twenty-one primary clinical studies met the inclusion criteria and were included in the review. Several studies demonstrated faster resolution of obstruction, shorter hospital stays, and lower rates of surgery. However, other studies did not show significant improvements in these outcomes. These discrepancies are likely due to differences in study design, patient selection, timing of contrast administration, and variations in WSC challenge protocols. In contrast, the prognostic value of WSC was consistent across studies, as the progression of contrast to the colon was reliably associated with successful nonoperative management. Overall, the evidence suggests that the primary value of WSC lies in its diagnostic and prognostic roles, while its therapeutic benefit remains uncertain.

## Introduction and background

Small bowel obstruction (SBO) is among the most frequent causes of emergency general surgery admission, accounting for approximately 20% of surgical emergency department visits for abdominal pain in Western countries, and is a major source of morbidity, recurrent hospitalization, and resource utilization [1-4]. Postoperative adhesions are the dominant cause of SBO in adults, making adhesive small bowel obstruction (ASBO) a recurring clinical problem for surgeons and emergency departments [1-5]. Although most uncomplicated adhesive obstructions can be managed nonoperatively, a subset of patients requires operative adhesiolysis or bowel resection [5,6]. One of the challenges in the management of ASBO is distinguishing patients who can be safely managed nonoperatively from those who would benefit from earlier surgical intervention [1,2,7-9]. Initial management of uncomplicated SBO typically consists of bowel rest, nasogastric decompression when indicated, intravenous fluid resuscitation, electrolyte correction, serial abdominal examinations, and radiographic assessment [1,10]. Surgical intervention is indicated when there are signs of strangulation, peritonitis, bowel ischemia, closed-loop obstruction, perforation, or failure of nonoperative management [1,10]. Prolonged delays in operative management after failed conservative treatment may be associated with increased morbidity and worse clinical outcomes [11]. However, unnecessary operative intervention exposes patients to increased morbidity, including infectious, cardiovascular, and postoperative complications [12].

Water-soluble contrast (WSC) agents are used as an adjunct during nonoperative management and have been incorporated into SBO management pathways for two reasons [1,13]. First, they can help predict which patients are likely to improve without surgery, as passage of contrast into the colon is associated with successful nonoperative management [13]. Second, these high-osmolar contrast agents may have a therapeutic effect by drawing fluid into the intestinal lumen, decreasing bowel wall edema, and promoting intestinal motility [14]. However, whether these physiologic effects translate into meaningful therapeutic benefit remains controversial.

Evidence regarding the therapeutic effects of Gastrografin and Urografin has been mixed. Early randomized trials and prospective cohort studies reported faster resolution of obstruction, shorter hospital stays, and lower rates of surgery, whereas subsequent randomized trials reported less consistent benefits [7,8,14-18]. Later meta-analyses likewise questioned whether these findings reflect a true therapeutic effect or improved clinical decision-making [8,19-21]. This distinction affects how WSC is used in clinical practice. If WSC's role is mainly diagnostic, it helps guide management. If its role is therapeutic, it may also improve patient outcomes. This review aims to synthesize the available evidence on WSC in SBO and clarify its clinical role. 

## Review

Methods

Search Strategy

A literature search was conducted in PubMed and the Excerpta Medica database (Embase) from database inception through June 2026. A manual search of the reference lists of eligible articles and relevant reviews was also performed to identify additional studies. The search was limited to English-language publications involving adult patients.

Search terms included combinations of "small bowel obstruction," "adhesive small bowel obstruction," "postoperative small bowel obstruction," "SBO," "ASBO," "Gastrografin," "Urografin," "water-soluble contrast," "diatrizoate," "Telebrix," "contrast challenge," "nonoperative management," "operative intervention," and "length of stay." The search focused on studies evaluating adult patients with ASBO managed with oral or nasogastric administration of WSC agents. 

Eligibility Criteria

Included studies evaluated adult patients (≥18 years) with adhesive, postoperative, non-strangulated, or uncomplicated SBO; evaluated oral or nasogastric administration of a WSC agent; and reported clinical outcomes such as contrast passage, nonoperative resolution, time to symptom resolution, operative intervention, bowel resection, length of stay, recurrence, or complications.

Randomized controlled trials, prospective studies, retrospective cohort studies, case reports, and protocol implementation studies were eligible for inclusion. Pediatric-only studies, animal studies, review articles, meta-analyses, technical imaging reports without clinical outcomes, and studies evaluating unrelated contrast agents were excluded.

Study Selection and Data Extraction

The literature search and study selection were performed in accordance with the PRISMA 2020 reporting guideline [22]. Duplicate records were removed, after which titles and abstracts were screened for relevance. Full-text articles were subsequently reviewed for eligibility based on the predefined inclusion and exclusion criteria. Reasons for exclusion during full-text review included pediatric populations, absence of relevant clinical outcomes, review articles, and unrelated study topics.

The following information was extracted from each study: study design, publication year, sample size, patient population, WSC protocol, comparator group, operative rate, length of stay, diagnostic performance when available, complications, and principal findings.

**Figure 1:**
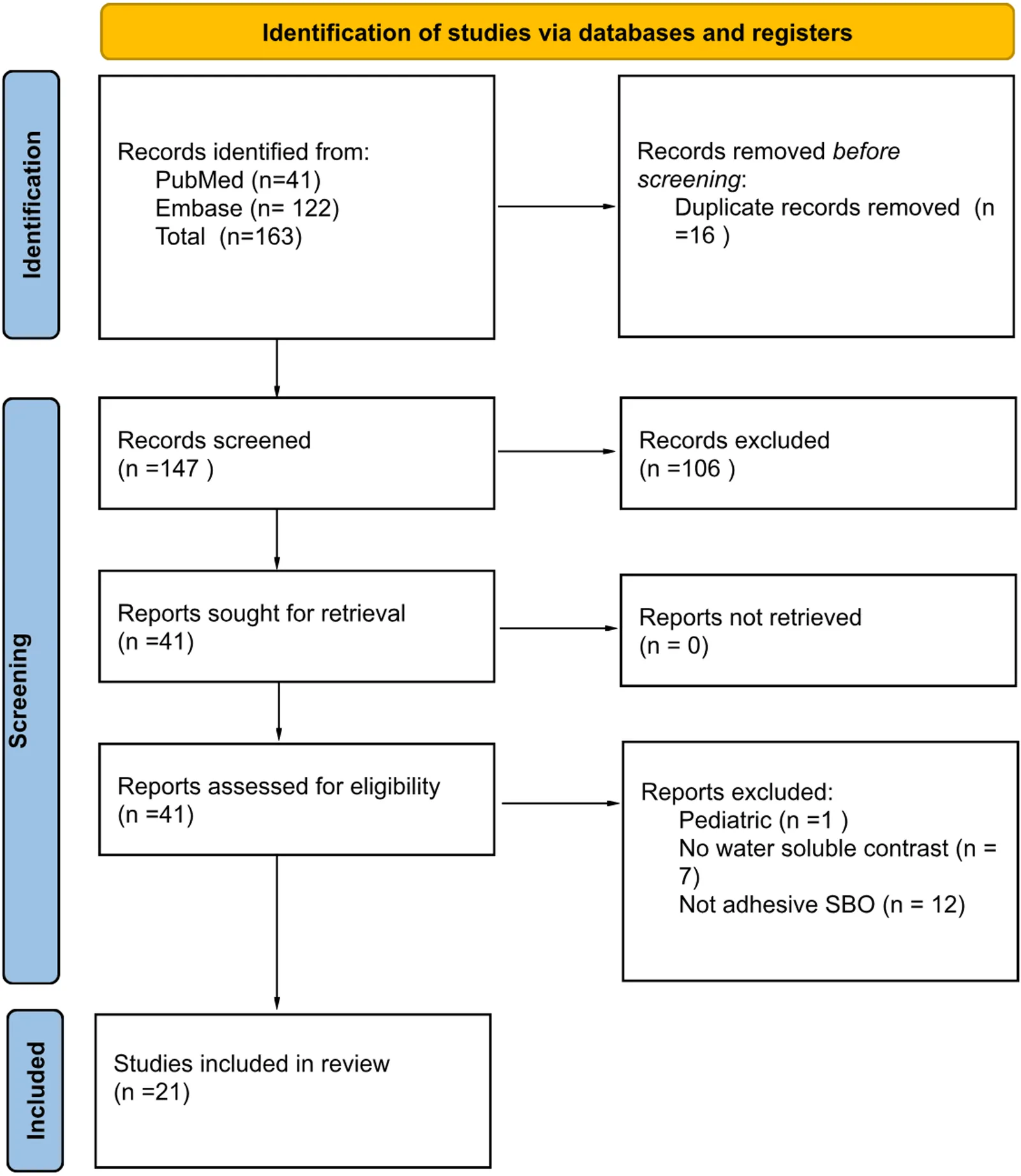
PRISMA 2020 flow diagram of the literature search and study selection process. Embase: Excerpta Medica database; SBO: small bowel obstruction

Given the clinical and methodological heterogeneity among the included studies, such as differences in study design, patient populations, WSC protocols, outcome definitions, and indications for surgery, a quantitative meta-analysis was not appropriate. Therefore, the findings were synthesized narratively by comparing the major clinical outcomes reported across the included studies. The study selection process is summarized in the PRISMA 2020 flow diagram (Figure 1).

Risk of Bias and Evidence Assessment

Because the objective of this study was to provide a narrative synthesis of the available evidence, a formal risk-of-bias assessment was not performed. Potential sources of bias, including selection bias, confounding, differences in institutional practice, and operative thresholds, were considered when interpreting the findings.

Results

Study Characteristics

Twenty-one studies published between 1996 and 2026 met the inclusion criteria [3,5-8,14-18,23-33]. The studies primarily included prospective randomized controlled trials, prospective cohort studies, observational studies, and protocol evaluation studies, with sample sizes varying from 41 to 601 patients. Most evaluated adults with adhesive or postoperative small bowel obstruction were managed nonoperatively. In contrast, a smaller number included patients with non-strangulated acute small bowel obstruction or obstruction following colorectal surgery. Gastrografin was the most commonly studied WSC agent, although Urografin, Telebrix Gastro, and meglumine ioxitalamate were also evaluated. The main outcomes included successful nonoperative management, surgical intervention, time to resolution, hospital length of stay, diagnostic performance of contrast passage, and predictors of failure of conservative treatment. Study characteristics are summarized in Table 1.

**Table 1:** Characteristics of the included studies. ASBO: adhesive small bowel obstruction; GG: Gastrografin; LOS: length of stay; NGT: nasogastric tube; POSBO: postoperative small bowel obstruction; RCT: randomized controlled trial; SBO: Small bowel obstruction; WSC: water-soluble contrast; h: hours

No.	Study	Study Design	Population	Patients (n)	Episodes (n)	Intervention/Comparator
1	Scotté et al., 2017 [8]	Multicenter open-label RCT	Patients with uncomplicated acute ASBO	242	—	100 mL oral GG after NGT decompression vs 100 mL saline
2	Burge et al., 2005 [25]	Double-blind RCT	Patients with ASBO eligible for conservative management	45 (43 analyzed)	—	GG via NGT vs. placebo
3	Moskowitz et al., 2019 [30]	Multicenter protocol evaluation	Patients with SBO managed at tertiary care centers	283	—	Standardized WSC challenge protocol; no randomized comparator
4	Choi et al., 2005 [14]	Prospective evaluation	Patients with ASBO after failed initial conservative management	212	245	100 mL GG after 48 h failed conservative management, no comparator
5	Bueno-Lledó et al., 2016 [6]	Prospective cohort	Patients with ASBO managed with a GG protocol	223	235	GG protocol; no comparator
6	Feigin et al., 1996 [23]	Prospective RCT	Patients with POSBO eligible for conservative management	50	—	100 mL meglumine ioxitalamate via NGT vs. standard conservative management
7	Miquel et al., 2017 [29]	Prospective cohort	Patients with ASBO after colorectal surgery	187	—	GG with 24-h radiograph; no comparator
8	Kuehn et al., 2017 [3]	Prospective controlled algorithm study	Patients with non-strangulated SBO	181	—	Algorithm incorporating contrast swallow/GG challenge vs. immediate surgery when indicated
9	Zielinski et al., 2017 [32]	Multicenter prospective observational study	Patients with ASBO	316	—	GG protocol vs. standard care without GG
10	Farid et al., 2010 [15]	Prospective RCT	Patients with ASBO	110	—	100 mL GG via NGT with 24-h radiograph vs. conservative management
11	Kumar et al., 2009 [18]	Prospective RCT	Patients with POSBO	41	—	Oral WSC agent vs. conventional conservative management
12	Collom et al., 2018 [5]	Prospective multicenter observational study	Patients with SBO with or without previous abdominal surgery	601	—	GG challenge vs. no GG
13	Choi et al., 2002 [31]	Prospective randomized trial	Patients with ASBO after failed conservative management	124	139	GG meal and follow-through after failed conservative management vs. immediate surgery
14	Biondo et al., 2003 [7]	Prospective RCT	Patients with ASBO	83	90	100 mL oral Gastrografin with 24-h radiograph vs. conservative management
15	Fevang et al., 2000 [24]	Prospective randomized trial	Patients with SBO	98	—	Upper GI contrast with barium plus sodium diatrizoate/GG vs. no contrast
16	Aulin et al., 2005 [28]	Prospective case series	Patients with ASBO undergoing a Telebrix Gastro challenge	97	126	100 mL Telebrix Gastro via gastric tube with 8-h radiograph; no comparator
17	Chen et al., 1999 [16]	Prospective study	Patients with POSBO without signs of bowel ischemia or strangulation	116	—	Oral/NG WSC (Urografin) with serial radiographs; no comparator
18	Katano et al., 2020 [27]	Multicenter RCT	Patients with non-strangulated acute SBO	223	—	NG tube with GG enterography vs. long tube decompression
19	Zhang et al., 2006 [26]	Randomized clinical trial	Older adults with ASBO	162	—	WSC (methylglucamine diatrizoate) plus octreotide vs. conservative management
20	Yagci et al., 2005 [17]	Prospective nonrandomized study	Patients with POSBO due to adhesions	317	338	WSC (Urografin) via NG tube vs. standard NG decompression
21	Jaanimäe et al., 2026 [33]	Prospective randomized pilot trial	Adhesive and virgin abdomen SBO	128	—	100 mL isotonic WSC (Visipaque) administered 4 h vs. 24 h after admission

Therapeutic Outcome Studies

The following studies evaluated the therapeutic effect of WSC compared with standard management. The primary outcomes included operative intervention, time to resolution, hospital length of stay, morbidity, and mortality.

Feigin et al. conducted a prospective randomized controlled trial involving 50 patients with postoperative SBO (POSBO) who were suitable for conservative management. Patients received 100 mL of meglumine ioxitalamate compared with standard conservative therapy. There were no significant differences in operative intervention rates (3/25 vs. 4/25), symptom resolution time (25.7 vs. 28.7 hours), hospital length of stay, or complications between groups. They concluded that WSC did not provide a therapeutic benefit but was safe and not associated with contrast-related complications [23].

Fevang et al. conducted a prospective randomized trial in 98 patients with SBO. Patients received a mixture of barium and Gastrografin or standard conservative management without contrast. No significant differences were observed in nonoperative resolution (31/48 vs. 35/50), strangulated bowel (1/48 vs. 4/50), bowel resection (3/48 vs. 4/50), complications (8/48 vs. 5/50), mortality (3/48 vs. 1/50), or hospital length of stay. However, patients receiving contrast underwent surgery earlier when operative intervention was required, suggesting that contrast may facilitate earlier surgical decision-making rather than improve obstruction resolution [24]. 

Biondo et al. conducted a prospective randomized controlled trial involving 83 patients (90 episodes) of postoperative ASBO. Patients received 100 mL of oral Gastrografin or conservative management. Conservative treatment was successful in 85.6% of episodes, whereas 14.4% required surgery. Although Gastrografin did not reduce operative intervention, it was associated with shorter hospital stays in both nonoperative (2.8 vs. 5.8 days) and operative patients (13.6 vs. 21.1 days) [7]. 

Burge et al. performed a double-blind randomized controlled trial in 45 patients with ASBO suitable for conservative management, of whom 43 were included in the final analysis. Patients received Gastrografin or a placebo. The authors observed that obstruction resolved more rapidly in the Gastrografin group (12 vs. 21 hours) and that hospital length of stay was shorter (three vs. four days). They found that Gastrografin accelerated the resolution of obstruction [25].

Yagci et al. conducted a prospective nonrandomized study involving 317 patients (338 episodes) of postoperative ASBO. Patients received Urografin or continuous nasogastric decompression alone. The Urografin group had higher rates of successful nonoperative management (89.4% vs. 75.4%), lower operative intervention (11.6% vs. 24.6%), faster recovery of bowel function, and a shorter hospital length of stay. These findings suggest that WSC may reduce the need for surgery and facilitate earlier operative decision-making [17].

Zhang et al. conducted a randomized clinical trial in 162 older adults with ASBO. Patients received methylglucamine diatrizoate plus octreotide or conservative management. The combination therapy was associated with lower pain scores, reduced nasogastric tube output, earlier recovery of bowel function, shorter hospital length of stay, lower rates of operative intervention, and reduced postoperative morbidity. The authors concluded that the combined regimen improved outcomes in older patients with ASBO [26].

Farid et al. conducted a prospective randomized controlled trial in 110 patients with ASBO. Patients received 100 mL of Gastrografin or conservative management alone. The Gastrografin group had lower rates of surgery (14.5% vs. 34.5%), faster resolution of obstruction (19.5 vs. 42.6 hours), and a shorter hospital length of stay (3.8 vs. 6.9 days). Gastrografin also accurately predicted the need for surgery, with a sensitivity of 87.5% and specificity of 100%, supporting both its therapeutic and diagnostic roles in ASBO [15]. 

Kumar et al. conducted a prospective randomized controlled trial including 41 patients with POSBO. Patients received an oral WSC agent compared with conventional management. The authors reported a shorter time to resolution of obstruction (7.47 vs. 35.20 hours) and reduced hospital length of stay among patients managed nonoperatively (3.43 vs. 5.33 days) in the contrast group. Operative intervention was required in seven of 21 patients in the contrast group and two of 20 in the conventional management group; however, this difference did not reach statistical significance. They concluded that WSC accelerated the resolution of POSBO and reduced the length of hospitalization [18]. 

The multicenter randomized controlled trial conducted by Katano et al. included 223 patients with non-strangulated acute SBO. Patients received nasogastric tube decompression with Gastrografin enterography compared with long-tube decompression. Investigators reported the nonoperative management rate was similar between groups (91.1% vs. 87.4%). No significant differences were observed in hospital length of stay or mortality. However, the Gastrografin strategy was a less invasive procedure with a shorter duration. They concluded that nasogastric tube decompression with Gastrografin enterography may be a practical alternative to long-tube decompression in patients with non-strangulated acute SBO [27]. 

Scotté et al. conducted a multicenter, open-label, randomized controlled trial involving 242 patients with uncomplicated acute ASBO. Patients received 100 mL of oral Gastrografin after nasogastric tube decompression compared with 100 mL of saline. The researchers noted no significant differences between groups in operative intervention rates (24% vs. 20%), bowel resection rates (8% vs. 4%), or hospital length of stay (3.8 vs. 3.5 days). They concluded that Gastrografin did not reduce the need for surgery or shorten hospitalization in uncomplicated ASBO [8]. 

A recently published randomized pilot trial also found that earlier administration of WSC (four hours versus 24 hours after admission) did not improve nonoperative resolution or reduce the need for surgery [33].

Diagnostic and Prognostic Studies

Five studies primarily evaluated the diagnostic and prognostic performance of WSC. Their primary outcomes included successful nonoperative management, the need for surgery, passage of contrast to the colon, and diagnostic accuracy measured by sensitivity, specificity, and predictive values.

The prospective study conducted by Chen et al. included 116 patients with POSBO without signs of bowel ischemia or strangulation. Patients received Urografin followed by serial abdominal radiographs. Contrast reached the colon within eight hours in 74 patients (63.8%), all of whom were successfully managed nonoperatively, whereas 34 of 42 patients (81.0%) without contrast passage required surgery. Urografin accurately distinguished partial from complete obstruction, with a sensitivity of 90.2%, specificity of 100%, and overall accuracy of 93.1% [16].

Aulin et al. conducted a prospective study with 97 patients and 126 episodes of ASBO. Patients received 100 mL of Telebrix Gastro, followed by abdominal radiographs 8 hours later. Contrast reached the colon in 113 of 126 episodes (89.7%); of these, 111 (98.2%) were successfully managed nonoperatively, whereas all 13 episodes (10.3%) without colonic contrast passage required surgery. The authors concluded that Telebrix Gastro accurately predicted successful conservative management, with a sensitivity of 98%, specificity of 100%, and overall accuracy of 98% [28].

Kühn et al. conducted a prospective, controlled study of 181 patients with SBO who were managed using a standardized treatment algorithm. Patients underwent a Gastrografin challenge or immediate surgery if bowel ischemia, perforation, or peritonitis was suspected. Of the 105 patients who underwent the Gastrografin challenge, 85 (81.0%) were successfully managed nonoperatively, whereas 20 (19.0%) required surgery. The Gastrografin challenge accurately predicted the need for operative intervention, with a sensitivity of 100%, specificity of 96%, and overall accuracy of 96% [3].

Miquel et al. conducted a cohort study involving 187 episodes of ASBO following colorectal surgery. Patients underwent a Gastrografin challenge followed by a 24-hour abdominal radiograph. Successful nonoperative management was achieved in 171 episodes (91.4%), whereas 16 episodes (8.6%) required surgery. The Gastrografin challenge accurately predicted successful conservative management, with a sensitivity of 75%, specificity of 99%, positive predictive value of 92%, and negative predictive value of 98%, supporting its use after colorectal surgery [29]. 

The multicenter protocol evaluation conducted by Moskowitz et al. included 283 patients admitted with SBO, of whom 245 (86.6%) were eligible for a WSC challenge, and 197 (80.4%) underwent the protocol. Patients received a standardized WSC challenge following computed tomography and nasogastric decompression. Successful contrast passage was associated with a shorter hospital length of stay, whereas failure to reach the colon within 24 hours was strongly associated with operative intervention. The authors concluded that a standardized WSC protocol improves patient triage and facilitates earlier surgical decision-making [30].

Management Protocol Studies and Predictors of Failure

Five studies evaluated the role of WSC within management protocols and investigated factors associated with failure of conservative treatment. These studies focused on protocol implementation, rescue strategies after failed conservative management, and clinical predictors of operative intervention.

Choi et al. conducted a prospective randomized trial in 124 patients (139 episodes) of ASBO that failed 48 hours of conservative management. Patients received a Gastrografin meal and follow-through or immediate surgery. Among the 19 episodes treated with Gastrografin, 14 (73.7%) with partial obstruction resolved without surgery, whereas all five patients with complete obstruction required operative intervention. Overall, Gastrografin reduced the need for surgery by 74% and was associated with no contrast-related complications, supporting its use as an adjunct after failed conservative management [31].

In 2005, Choi et al. expanded this approach in a prospective study involving 212 patients (245 episodes) of ASBO after failed conservative management. Patients received 100 mL of Gastrografin after 48 hours of unsuccessful conservative treatment. Among the 44 episodes treated with Gastrografin, 37 (84.1%) had partial obstruction; of these, 36 (97.3%) resolved without surgery, whereas all seven patients with complete obstruction required operative intervention. The overall rate of surgery was 10%, and no Gastrografin-related complications were reported, further supporting the safety of Gastrografin as an adjunct after failed conservative treatment [14].

Bueno-Lledó et al. conducted a prospective cohort study including 223 patients and 235 episodes of ASBO managed with a Gastrografin protocol. Conservative management was successful in 198 of 235 episodes (84.2%), whereas 33 patients (15.8%) ultimately required surgery. Only three patients in whom contrast reached the colon subsequently underwent operative intervention. Increasing age, multiple previous laparotomies, and a history of surgery for ASBO were associated with nonoperative management failure. The authors suggested that these clinical characteristics may help identify patients who are less likely to benefit from conservative treatment with Gastrografin [6].

The prospective multi-institutional observational study performed by Zielinsky et al. included 316 patients with ASBO. Patients received a Gastrografin protocol or standard management without Gastrografin. The study demonstrated lower rates of operative exploration (20.8% vs. 44.1%), bowel resection (6.9% vs. 21.0%), and shorter hospital length of stay (four vs. five days) in the Gastrografin group, while complication rates were similar between groups (12.5% vs. 17.9%). Multivariable analysis showed that Gastrografin use was independently associated with successful nonoperative management. The authors concluded that a Gastrografin protocol was associated with lower exploration rates and shorter hospitalization [32].

Collom et al. conducted a prospective, multi-institutional observational study of 601 patients with SBO, including those with and without prior abdominal surgery. Patients underwent a Gastrografin challenge or standard management. Multivariable analysis showed that Gastrografin use was independently associated with successful nonoperative management. Among patients with previous abdominal surgery, operative exploration occurred in 16.9% of the Gastrografin group versus 46.2% of the standard management group. Similarly, among patients without previous abdominal surgery, operative exploration occurred in 21.8% and 40.0% of patients, respectively. The authors concluded that a Gastrografin challenge may be considered for selected patients, regardless of prior abdominal surgery [5]. 

Discussion

Therapeutic Role of WSC 

Early clinical studies suggested that WSC may provide therapeutic benefit in patients with adhesive small bowel obstruction by accelerating obstruction resolution and shortening hospital length of stay. Burge et al., Biondo et al., Farid et al., and Kumar et al. all reported faster resolution of obstruction and/or shorter hospitalization with WSC. However, improvements in operative intervention were inconsistent [7,15,18,25]. These findings are supported by earlier meta-analyses, which also demonstrated reductions in time to resolution and hospital length of stay but found no consistent reduction in the need for surgery [13,21]. 

However, more recent evidence has called into question the consistency of these therapeutic benefits. Feigin et al., Fevang et al., Katano et al., and Scotté et al. found no significant reductions in operative intervention or hospital length of stay compared with standard conservative management [8,23,24,27]. The contemporary meta-analysis by Koh et al. demonstrated persistent reductions in hospital stay but no convincing reduction in operative intervention [20]. Similarly, Jaanimäe et al. found that administering WSC 4 hours rather than 24 hours after admission did not improve nonoperative resolution or reduce the need for surgery [33].

Several factors may explain these conflicting findings. The included studies differed considerably in patient selection, with some enrolling only patients with uncomplicated ASBO suitable for conservative management [8,23,25], whereas others included patients with POSBO [7,18,23], older adults [26], or non-strangulated acute SBO [27]. The timing of WSC administration also varied substantially. Some investigators administered contrast soon after diagnosis [8,15,23], while others reserved it for patients who failed an initial 48-hour period of conservative management [14,31]. In addition, treatment protocols were not uniform. Zhang et al. combined WSC with octreotide, making it difficult to determine the independent effect of contrast [26], whereas Fevang et al. administered a mixture of barium and Gastrografin instead of Gastrografin alone [24]. Finally, many of the earlier randomized trials enrolled relatively small numbers of patients [18,23,25,29], increasing the risk of imprecision. These methodological differences likely contributed to the variability in reported therapeutic outcomes and have been highlighted in recent meta-analyses [19,20].

Overall, the available evidence suggests that WSC may shorten the time to resolution of obstruction and hospital length of stay in selected patients managed nonoperatively. However, its effect on reducing the need for surgery remains unclear. This is in line with the 2017 Bologna Guidelines, which recommend WSC as an adjunct to conservative management and as a tool to guide clinical decision-making, rather than as a treatment to avoid surgery [1]. The therapeutic outcomes of the above-mentioned studies are summarized in Table 2. 

**Table 2:** Therapeutic outcomes of WSC studies in small bowel obstruction. LOS: length of stay; NR: not reported; WSC: water-soluble contrast; d: days; h: hours. *Outcomes in Zhang et al. should be interpreted cautiously because patients received octreotide in addition to WSC.

Study	Surgery	Hospital LOS	Resolution of Obstruction	Main Finding
Feigin et al., 1996 [23]	No difference (3/25 vs. 4/25)	No difference	No difference (25.7 vs. 28.7 h)	No therapeutic benefit; safe
Fevang et al., 2000 [24]	No difference	No difference	No difference	Earlier surgery when required; no therapeutic benefit
Biondo et al., 2003 [7]	No difference	Shorter LOS (2.8 vs. 5.8 d; operative: 13.6 vs. 21.1 d)	NR	Shorter LOS only
Burge et al., 2005 [25]	No difference	Shorter LOS (3 vs. 4 d)	Faster resolution (12 vs. 21 h)	Faster resolution and shorter LOS
Yagci et al., 2005 [17]	Lower operative rate (11.6% vs. 24.6%)	Shorter LOS	Faster bowel recovery	Improved nonoperative success
Zhang et al., 2006* [26]	Lower operative rate	Shorter LOS	Earlier bowel function	Benefit observed with the combined WSC plus octreotide regimen
Kumar et al., 2009 [18]	No significant difference	Shorter LOS (3.43 vs. 5.33 d)	Faster resolution (7.5 vs. 35.2 h)	Faster resolution and shorter LOS
Farid et al., 2010 [15]	Lower operative rate (14.5% vs. 34.5%)	Shorter LOS (3.8 vs. 6.9 d)	Faster resolution (19.5 vs. 42.6 h)	Therapeutic and diagnostic benefit
Scotté et al., 2017 [8]	No difference (24% vs. 20%)	No difference (3.8 vs. 3.5 d)	No difference	No therapeutic benefit
Katano et al., 2020 [27]	No difference	No difference	Similar nonoperative success	Noninferior to long tube decompression
Jaanimäe et al., 2026 [33]	No difference	No difference	No difference	Earlier WSC administration showed no additional therapeutic benefit

Diagnostic and Prognostic Role of WSC

The diagnostic and prognostic role of WSC was more consistent across the included studies than its therapeutic role. Chen et al., Aulin et al., Kühn et al., Miquel et al., and Moskowitz et al. all demonstrated that contrast reaching the colon accurately predicted successful nonoperative management. In contrast, failure of contrast progression was strongly associated with the need for operative intervention [3,16,28-30]. Across these studies, contrast reached the colon within 24 hours in most patients who responded to conservative treatment, whereas failure of progression consistently predicted the need for surgery. Reported sensitivities ranged from 75% to 100%, and specificities from 96% to 100%, indicating excellent diagnostic performance across diverse patient populations and treatment protocols.

Multiple systematic reviews and meta-analyses support these findings. Branco et al. and Ceresoli et al. concluded that the greatest clinical value of WSC lies in its ability to predict the success or failure of conservative management rather than its therapeutic effect [13,21]. Similarly, Koh et al. reported that contrast reaching the colon within 24 hours was a reliable predictor of nonoperative resolution and emphasized its usefulness in guiding treatment decisions [20].

The prognostic value of WSC has important clinical implications. Identifying patients who are unlikely to respond to conservative management may reduce delays in operative intervention, shorten unnecessary observation periods, and optimize resource utilization. Rather than replacing clinical judgment, WSC should be considered an adjunct to serial abdominal examinations, laboratory findings, and cross-sectional imaging in determining the need for surgery [1,3,30]. The diagnostic and prognostic performance of WSC in SBO is summarized in Table 3.

**Table 3:** Diagnostic and prognostic performance of water-soluble contrast in SBO. LOS: length of stay; SBO: small bowel obstruction

Study	Diagnostic Criterion	Sensitivity	Specificity	Main Finding
Chen et al., 1999 [16]	Contrast reached the colon within eight hours	90.2%	100%	Accurately distinguished partial from complete SBO
Aulin et al., 2005 [28]	Contrast reached the colon within eight hours	98%	100%	Excellent predictor of successful nonoperative management
Kuehn et al., 2017 [3]	Gastrografin challenge algorithm	100%	96%	Accurately predicted the need for operative intervention
Miquel et al., 2017 [29]	Contrast reached the colon within 24 hours	75%	99%	Predicted successful nonoperative management after colorectal surgery
Moskowitz et al., 2019 [30]	Contrast passage within 24 hours	Not reported	Not reported	Successful passage was associated with shorter LOS; failure predicted surgery

Role of WSC in Clinical Management

Several studies evaluated how WSC can be incorporated into management protocols rather than its therapeutic or diagnostic performance alone. Choi et al. demonstrated that WSC may help identify patients who can safely continue conservative management after an initial 48-hour trial. In contrast, Bueno-Lledó et al. identified older age, multiple previous laparotomies, and prior surgery for ASBO as predictors of nonoperative treatment failure [6,14,31]. In addition, Zielinski et al. and Collom et al. reported lower rates of operative intervention and shorter hospital stays among patients managed with standardized Gastrografin protocols. However, both studies acknowledged the limitations of their observational designs [5,32].

These findings suggest that the greatest value of WSC may lie in its incorporation into structured management pathways rather than in its use as a standalone therapeutic intervention. By identifying patients likely to respond to conservative management and those who may require earlier surgery, standardized WSC protocols may improve patient selection and optimize clinical decision-making. This approach is also supported by the 2017 Bologna Guidelines, which recommend incorporating WSC into the nonoperative management of selected patients with adhesive SBO [1].

**Figure 2:**
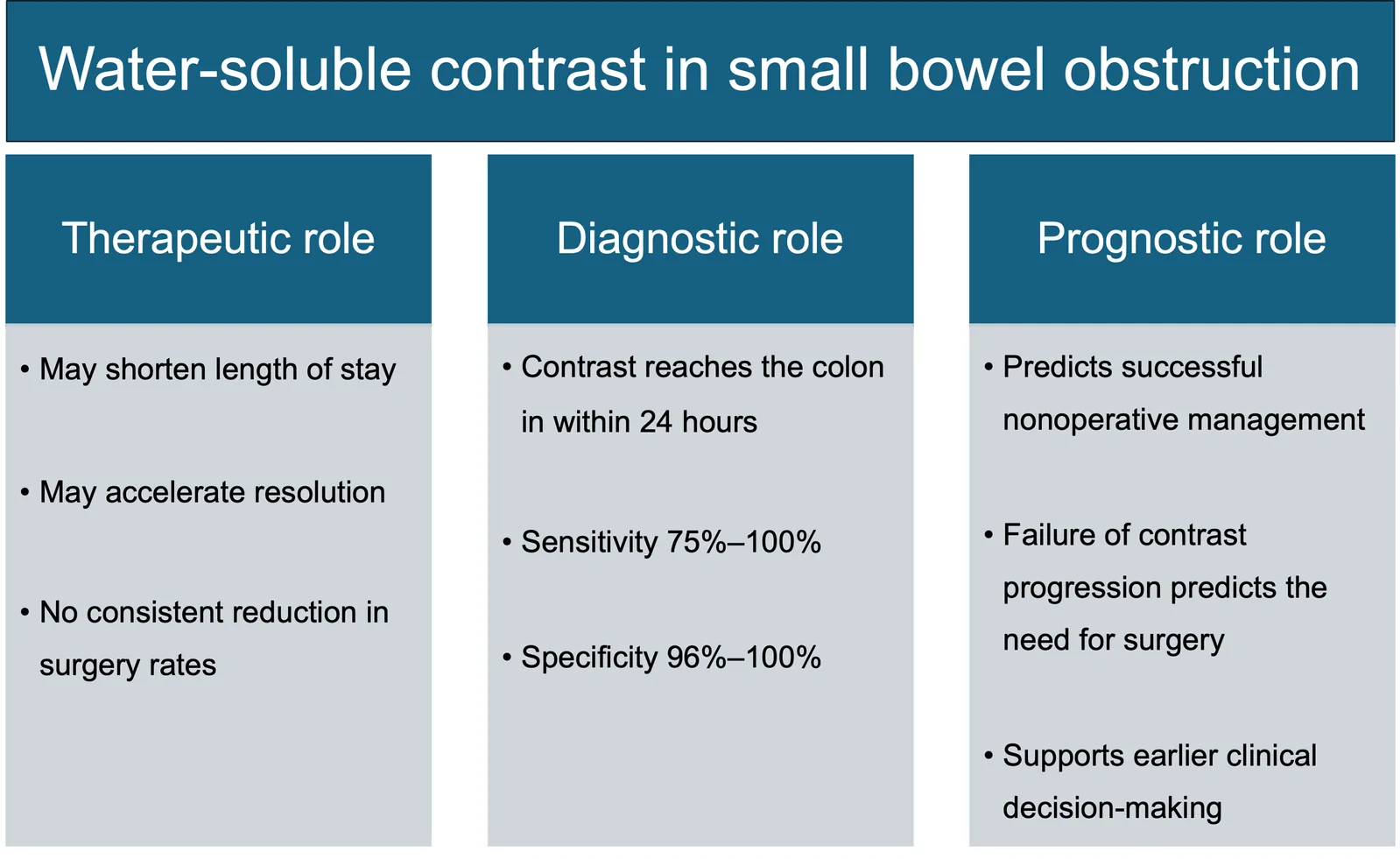
Summary of the diagnostic, prognostic, and therapeutic roles of water-soluble contrast in adhesive small bowel obstruction. Image credit: Puneet S. Gill; created using Microsoft PowerPoint (Microsoft Corp., Redmond, WA, USA). Content adapted from the findings of the studies included in this review [6-8,14-18,23-33].

The three roles of WSC in SBO discussed in this review are summarized in Figure 2. 

Limitations

This review has several limitations. The included studies differed in several respects, including study design, patient population, contrast agent, timing of WSC administration, and criteria for operative intervention, making direct comparison between studies difficult. Although several randomized controlled trials were included, many were single-center studies with relatively small sample sizes, while the observational studies were subject to selection bias and confounding. In addition, this review did not include a quantitative meta-analysis or a formal risk-of-bias assessment. Finally, only English-language studies indexed in the selected databases were included, which may have introduced publication and language bias. 

## Conclusions

SBO remains one of the most common surgical emergencies and a frequent cause of hospitalization and operative intervention. WSC plays an important role in the management of ASBO. Early randomized trials and prospective studies reported faster resolution of obstruction, shorter hospital stays, and, in some cases, lower operative rates with WSC. However, more recent randomized trials and subsequent meta-analyses have not consistently confirmed these therapeutic benefits. The differences between earlier and more recent studies likely reflect variations in patient populations, contrast administration protocols, timing of administration, and criteria for operative intervention. In contrast, the diagnostic and prognostic role of WSC has been consistently supported across studies. Progression of contrast to the colon has demonstrated high diagnostic accuracy for predicting successful nonoperative management, supporting its use as an adjunct to guide clinical decision-making. Further prospective studies using standardized protocols are needed to better define the therapeutic role of WSC and its impact on clinical outcomes.

## References

[1] Ten Broek RP, Krielen P, Di Saverio S (2018). Bologna guidelines for diagnosis and management of adhesive small bowel obstruction (ASBO): 2017 update of the evidence-based guidelines from the World Society of Emergency Surgery ASBO Working Group. World J Emerg Surg.

[2] Podda M, Khan M, Di Saverio S (2021). Adhesive small bowel obstruction and the six W’s: who, how, why, when, what, and where to diagnose and operate?. Scand J Surg.

[3] Kuehn F, Weinrich M, Ehmann S, Kloker K, Pergolini I, Klar E (2017). Defining the need for surgery in small-bowel obstruction. J Gastrointest Surg.

[4] Sakari T, Sköldberg F, Dietrich CE, Nordenvall C, Karlbom U (2024). Incidence of adhesive small bowel obstruction after surgery for colorectal cancer in Sweden 2007-2016. Colorectal Dis.

[5] Collom ML, Duane TM, Campbell-Furtick M (2018). Deconstructing dogma: nonoperative management of small bowel obstruction in the virgin abdomen. J Trauma Acute Care Surg.

[6] Bueno-Lledó J, Barber S, Vaqué J, Frasson M, Garcia-Granero E, Juan-Burgueño M (2016). Adhesive small bowel obstruction: predictive factors of lack of response in conservative management with Gastrografin. Dig Surg.

[7] Biondo S, Parés D, Mora L, Martí Ragué J, Kreisler E, Jaurrieta E (2003). Randomized clinical study of Gastrografin administration in patients with adhesive small bowel obstruction. Br J Surg.

[8] Scotté M, Mauvais F, Bubenheim M (2017). Use of water-soluble contrast medium (gastrografin) does not decrease the need for operative intervention nor the duration of hospital stay in uncomplicated acute adhesive small bowel obstruction? A multicenter, randomized, clinical trial (Adhesive Small Bowel Obstruction Study) and systematic review. Surgery.

[9] Kaplan LJ, Martinez-Casas I, Mohseni S, Cimino M, Kurihara H, Lee MJ, Bass GA (2025). Small bowel obstruction outcomes according to compliance with the World Society of Emergency Surgery Bologna guidelines. Br J Surg.

[10] Ghimire P, Maharjan S (2023). Adhesive small bowel obstruction: a review. JNMA J Nepal Med Assoc.

[11] Schraufnagel D, Rajaee S, Millham FH (2013). How many sunsets? Timing of surgery in adhesive small bowel obstruction: a study of the Nationwide Inpatient Sample. J Trauma Acute Care Surg.

[12] Fevang BT, Jensen D, Svanes K, Viste A (2002). Early operation or conservative management of patients with small bowel obstruction?. Eur J Surg.

[13] Branco BC, Barmparas G, Schnüriger B, Inaba K, Chan LS, Demetriades D (2010). Systematic review and meta-analysis of the diagnostic and therapeutic role of water-soluble contrast agent in adhesive small bowel obstruction. Br J Surg.

[14] Choi HK, Law WL, Ho JW, Chu KW (2005). Value of gastrografin in adhesive small bowel obstruction after unsuccessful conservative treatment: a prospective evaluation. World J Gastroenterol.

[15] Farid M, Fikry A, El Nakeeb A, Fouda E, Elmetwally T, Yousef M, Omar W (2010). Clinical impacts of oral gastrografin follow-through in adhesive small bowel obstruction (SBO). J Surg Res.

[16] Chen SC, Chang KJ, Lee PH, Wang SM, Chen KM, Lin FY (1999). Oral urografin in postoperative small bowel obstruction. World J Surg.

[17] Yagci G, Kaymakcioglu N, Can MF, Peker Y, Cetiner S, Tufan T (2005). Comparison of Urografin versus standard therapy in postoperative small bowel obstruction. J Invest Surg.

[18] Kumar P, Kaman L, Singh G, Singh R (2009). Therapeutic role of oral water soluble iodinated contrast agent in postoperative small bowel obstruction. Singapore Med J.

[19] Gowell M, Baker DM, McLachlan G, Naumann DN, Peckham-Cooper A, Smart NJ, Lee MJ (2025). Water-soluble contrast agents in adhesional small bowel obstruction: meta-analysis and PRECIS-2 assessment of trials. BJS Open.

[20] Koh A, Adiamah A, Chowdhury A, Mohiuddin MK, Bharathan B (2020). Therapeutic role of water-soluble contrast media in adhesive small bowel obstruction: a systematic review and meta-analysis. J Gastrointest Surg.

[21] Ceresoli M, Coccolini F, Catena F, Montori G, Di Saverio S, Sartelli M, Ansaloni L (2016). Water-soluble contrast agent in adhesive small bowel obstruction: a systematic review and meta-analysis of diagnostic and therapeutic value. Am J Surg.

[22] Page MJ, McKenzie JE, Bossuyt PM (2021). The PRISMA 2020 statement: an updated guideline for reporting systematic reviews. BMJ.

[23] Feigin E, Seror D, Szold A (1996). Water-soluble contrast material has no therapeutic effect on postoperative small-bowel obstruction: results of a prospective, randomized clinical trial. Am J Surg.

[24] Fevang BT, Jensen D, Fevang J (2000). Upper gastrointestinal contrast study in the management of small bowel obstruction-a prospective randomised study. Eur J Surg.

[25] Burge J, Abbas SM, Roadley G, Donald J, Connolly A, Bissett IP, Hill AG (2005). Randomized controlled trial of Gastrografin in adhesive small bowel obstruction. ANZ J Surg.

[26] Zhang Y, Gao Y, Ma Q (2006). Randomised clinical trial investigating the effects of combined administration of octreotide and methylglucamine diatrizoate in the older persons with adhesive small bowel obstruction. Dig Liver Dis.

[27] Katano T, Shimura T, Nishie H (2020). The first management using intubation of a nasogastric tube with Gastrografin enterography or long tube for non-strangulated acute small bowel obstruction: a multicenter, randomized controlled trial. J Gastroenterol.

[28] Aulin A, Sales JP, Bachar S, Hennequin J, Moumouh A, Tasu JP (2005). Telebrix Gastro in the management of adhesive small bowel obstruction. Gastroenterol Clin Biol.

[29] Miquel J, Biondo S, Kreisler E, Uribe C, Trenti L (2017). Failure of conservative treatment with Gastrografin® for adhesive small bowel obstruction after colorectal surgery. Int J Colorectal Dis.

[30] Moskowitz EE, McIntyre RC, Burlew CC (2019). Evaluation of a water-soluble contrast protocol for small bowel obstruction: a southwestern surgical congress multicenter trial. Am J Surg.

[31] Choi HK, Chu KW, Law WL (2002). Therapeutic value of gastrografin in adhesive small bowel obstruction after unsuccessful conservative treatment: a prospective randomized trial. Ann Surg.

[32] Zielinski MD, Haddad NN, Cullinane DC (2017). Multi-institutional, prospective, observational study comparing the Gastrografin challenge versus standard treatment in adhesive small bowel obstruction. J Trauma Acute Care Surg.

[33] Jaanimäe L, Lepner U, Kirsimägi Ü, Saarevet V, Nikkolo C (2026). A prospective randomised pilot study on the timing of contrast media administration in adhesive and virgin abdomen small bowel obstruction. Medicina (Kaunas).

